# Effects of Tibetan Music on Neuroendocrine and Autonomic Functions in Patients Waiting for Surgery: A Randomized, Controlled Study

**DOI:** 10.1155/2018/9683780

**Published:** 2018-03-05

**Authors:** Antonella Cotoia, Floriana Dibello, Fiorenzo Moscatelli, Alberto Sciusco, Pietro Polito, Alberto Modolo, Crescenzio Gallo, Giuseppe Cibelli, Gilda Cinnella

**Affiliations:** ^1^Department of Anesthesia, Intensive Care, and Pain Therapy, University of Foggia, Policlinico “OO. Riuniti”, Foggia, Italy; ^2^Department of Clinical and Experimental Medicine, University of Foggia, Policlinico “OO. Riuniti”, Foggia, Italy; ^3^Department of Intensive Care and Anaesthesia, North Bristol NHS Trust, Bristol, UK; ^4^Department of Blood Transfusion Medicine, ASS 5 Friuli Occidentale, Pordenone, Italy; ^5^Naturopathy, A.N.E.A. Academy, Prato, Italy

## Abstract

**Background:**

The aim of this study was to investigate the effects of listening to Tibetan music on anxiety and endocrine, autonomic, cognitive responses in patients waiting for urologic surgery.

**Methods:**

Sixty patients waiting for surgery were enrolled to the study. They were randomized in music (M) and control (C) groups. The M group listened to a low-frequency Tibetan music for 30 min (T_0_–T_30_) through headphones, and the C group wore headphones with no sound. The State Trait Anxiety Inventory Questionnaire (STAI) Y-1 was administered at T_0_ and T_30_. Normalized low (LFnu) and high frequencies (HFnu) of heart rate variability, LF/HF ratio, and galvanic skin response (GRS) data were analyzed at T_0_, T_10_, T_20_, T_30_, and T_35_. The salivary *α*-amylase (sAA) samples were collected at T_0_, T_35_, and T_45_.

**Results:**

In the M group, the STAI Y-1 score decreased at T_30_ versus baseline (*p* < 0.001), sAA levels decreased at T_35_ versus T_0_(*p*=0.004), and GSR remained unchanged. In the C group, the STAI Y-1 score remained unchanged, sAA level increased at T_35_ versus T_0_(*p* < 0.001), and GSR slightly increased at T_35_ versus baseline (*p*=0.359). LFnu was lower, and HFnu was significantly higher (T_10_–T_30_) in M versus C group. Mean LF/HF ratio slightly reduced in the M group.

**Conclusions:**

Our results suggest that preoperative listening to relaxing Tibetan music might be a useful strategy to manage preoperative anxiety.

## 1. Introduction

Preoperative anxiety is an emotional state characterized by feelings of tension, fear, nervousness, and apprehension which is common in patients waiting for surgery [1, 2]. The experience of anxiety and psychological stress before surgery is a complex phenomenon that comprises both physiological and psychological components and might require preoperative treatment [3].

The physiological response to stress involves the activation of the hypothalamic-pituitary-adrenal axis and the autonomic nervous system (ANS) through modulation of its branches, sympathetic and parasympathetic [4, 5], which can be evaluated by biochemical and physiological measurements, including salivary alpha-amylase (sAA) levels, heart rate variability (HRV), and galvanic skin response (GSR) [6–9].

Music is attracting increasing interest as a nonpharmaceutical therapeutic alternative for the treatment of preoperative anxiety in adult patients. Previous investigations found that music was effective in reducing the perceived levels of psychological stress and the need for additional analgesic doses [10, 11]. Other studies focused on physiological anxiolytic effects of music in the clinical setting [12, 13]. However, a clear understanding has not yet emerged regarding which genre of music is most beneficial to patients [14]. The perception of music as relaxing may vary in each patient, and the selection of the preferred music is suggested [15]. The most common notion is that the self-chosen music involves patient's mind with something familiar and restful, focusing the patient's attention away from negative stimuli to something pleasant and encouraging [11, 14]. Some music therapists prefer classical music for relaxation because the musical pieces are consistent [16]. Conversely, a study showed that melodies with elements of natural sound had positive effects in relation to patient's discomfort and pain [17].

We hypothesized that a relaxing music with low frequency has itself an effect on anxiety levels where it is listened for the first time and it is not self-selected. The aim of this study was to investigate the cognitive and emotional responses of listening to Tibetan relaxing music preoperatively and its effects on the endocrine system and ANS, in patients waiting for urologic surgery.

## 2. Materials and Methods

This prospective randomized, double-blinded study was performed in the Urologic Department of University Hospital of Foggia, Italy, from April 2017 to June 2017. Ethical approval for this study (Ethical Committee N° 5/CE/2016) was provided by the Ethical Committee of the University Hospital of Foggia on 14 February 2016. Consecutive patients between 45 and 65 years, undergoing elective major urologic surgery, and with American Society of Anesthesiologists (ASA) physical status of class II-III, were considered for enrollment. Prior to data collection, the purpose of this study was carefully explained and written informed consent was obtained from each participant, according to the Declaration of Helsinki.

The sound of Tibetan Bowls, invented and played by Alberto Modolo, was found in a range from 0 to 1 kHz by the time-frequency analysis (Mathematica software, Version 11.0, Wolfram Research Inc.) via short-time Fourier transform (Supplementary Material S1).

Patients playing any musical instrument, smokers, having hearing impairment, endocrine disorders, any known psychiatric or neurologic disorders, or taking any medication affecting ANS were excluded.

### 2.1. Measures

Quantitative measurements of anxiety in both physiological and psychological aspects were included.

#### 2.1.1. State Trait Anxiety Inventory Questionnaire (STAI)

The STAI consists in 40 items to measure the presence and severity of preoperative anxiety (STAI Y-1) and generalized propensity to be anxious (STAI Y-2). Total score obtained from each STAI range from 20 to 80, and the higher score indicates greater anxiety [18, 19]. A cut point of 39-40 has been suggested to detect clinically significant symptoms for the STAI scales [20, 21].

#### 2.1.2. Amsterdam Preoperative Anxiety and Information Scale (APAIS)

The APAIS is a 6-item questionnaire created to identify anxious patients (4 items to investigate on fear of anesthesia and the surgical procedure) and their need for information (2 items) [22].

The measurement for each item is rated on a 5-point Likert scale, where point 1 matches ‘not at all' and point 5 represents the ‘extremely anxiety' (total range 6–30).

The cutoff score for the anxiety scale was ≥10 for the whole sample [23].

#### 2.1.3. Heart Rate Variability (HRV)

Heart rate variability (HRV) is the physiological phenomenon of variation in the time interval between heartbeats, and it can be measured with frequency analysis of the ECG. Low frequency (LF, 0.04–0.15 Hz) reflects the combination of sympathetic and parasympathetic ANS modulation; high frequency (HF, 0.15–0.40 Hz) is a measure of vagal modulation on the heart; the LF/HF ratio is used, not without some controversy, to quantify the degree of sympathovagal balance [24].

The patients' HRV was measured with the Faros device (eMotion Faros 90°, Mega Electronics Ltd, Finland) whose sensor, placed on the right midclavicular line, was attached to the cable connected to the ECG electrode placed on the lower left abdomen within the rib cage frame. After recording was over, the data were transferred to computer, and we used Kubios HRV software (Kubios HRV, Version 2.2, http://kubios.uef.fi) to perform the HRV analysis. The powers of LF and HF bands in normalized units were considered for the data analysis (LFnu and HFnu) [25].

#### 2.1.4. Galvanic Skin Response (GSR)

We also monitored the emotional state by GSR, which originates from the autonomic activation of sweat glands in the skin. An increase in the stress level will cause sweat and a decreased skin resistance, resulting in measurable increased skin conductance by the balance of positive and negative ions in the secreted fluid.

The GSR parameters were measured using the SenseWear Pro Armband™ (Version 3.0, Body Media, Inc., PA, USA), which was worn on the right arm over the triceps muscle at the midpoint between the acromion and olecranon processes, as recommended by the manufacturer [26].

#### 2.1.5. Salivary Alpha-Amylase (sAA) Kinetic Enzyme Assay

We measured the sAA levels whose secretion was controlled by the autonomic nervous signals.

Salivary samples were collected with special sampling tubes (Salivette, Sarstedt, Numbrecht, Germany), and sAA were assayed by Salimetrics® *α*-Amylase Kinetic Enzyme Assay Kit following the standard guidelines outlined in the manual provided by the manufacturer Salimetrics Inc. [27].

### 2.2. Procedure

Sampling was performed between 8 : 00 and 11 : 00 am in a quiet room of the urologic ward before arrival in the operating room. Patients were randomized via computer-generated assignment to 2 groups: the music group (M) and the control group (C).

No sedatives were given in any groups. The M group listened to the Tibetan soundtrack for 30 min through headphones that covered the whole ear so that no external noise could interfere; the C group wore headphones for 30 min with no sound.

Before the application of the headphones (T_0_), we collected STAI Y-1 and STAI Y-2 scores, APAIS questionnaires score, and sAA samples.

After removal of the headphones (T_30_), we collected STAI Y-1 score.

Additionally, sAA samples were obtained at 5 min and 15 min after the headphones removal (T_35_ and T_45_).

**Figure 1:**
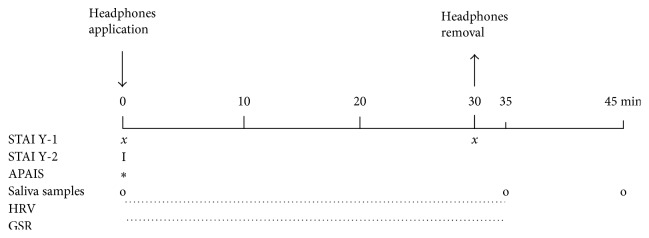
Timeline of the study procedure: State Trait Anxiety Inventory Questionnaire (STAI); Amsterdam Preoperative Anxiety and Information Scale (APAIS); heart rate variability (HRV); galvanic skin response (GSR).

The HRV and GSR data were analyzed at T_0_, T_10_, T_20_, T_30,_ and T_35_ (Figure 1).

### 2.3. Statistical Analysis

On the basis of previous investigations, a sample size of 22 subjects per group was able to detect a normalized LF decrease of 8.5% in stress-induced students after listening music for 20 min (assuming *α* = 0.05 and power = 0.95) [28]. This number was increased to 30 per group to allow for a 35% patients dropout rate.

The normality of distribution was assessed by Shapiro-Wilkinson test. Since we found all of the data normally distributed, the data were expressed as mean ± SD. Data were analyzed using one-ways ANOVA and repeated measurement analysis of variance. The Bonferroni multiple comparison test was performed to identify significant differences among measures. Differences between the groups at each time point were examined post hoc using independent sample *t*-test. A paired sample *t*-test was used to detect changes within the groups. A value of *p* < 0.05 was considered statistically significant. Statistical analysis was performed by Statistical Package for the Social Sciences (SPSS Inc., Chicago, IL) Version 15.0 for Windows.

## 3. Results

**Figure 2:**
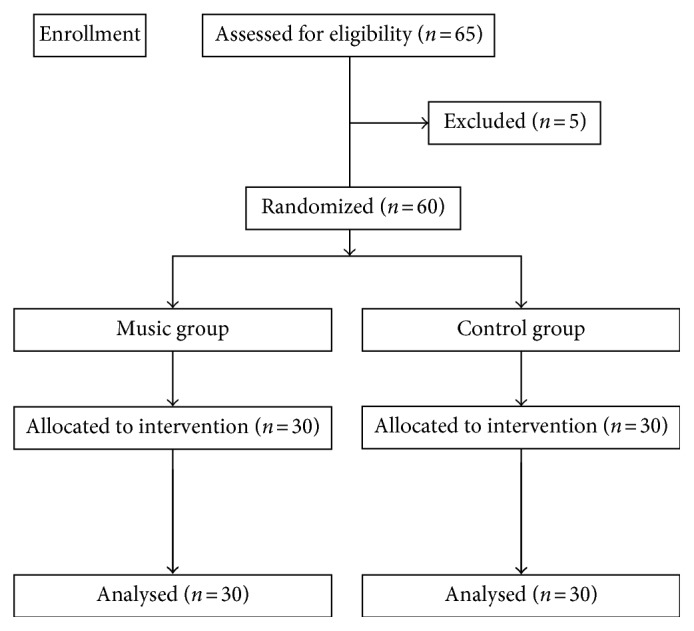
Flowchart of patients' enrollment.

The enrollment flow diagram is reported in Figure 2. 60 out of 65 candidates for enrollment were included in the study and were randomly divided into two groups: 30 patients (15 males and 15 females) in the “Music group” (M) and 30 patients (15 males and 15 females) in the “Control group” (C).

There were no differences between the two groups as regards for age, sex, ASA, and types of surgical procedures performed (Table 1).

### 3.1. Psychological Tests

**Figure 3:**
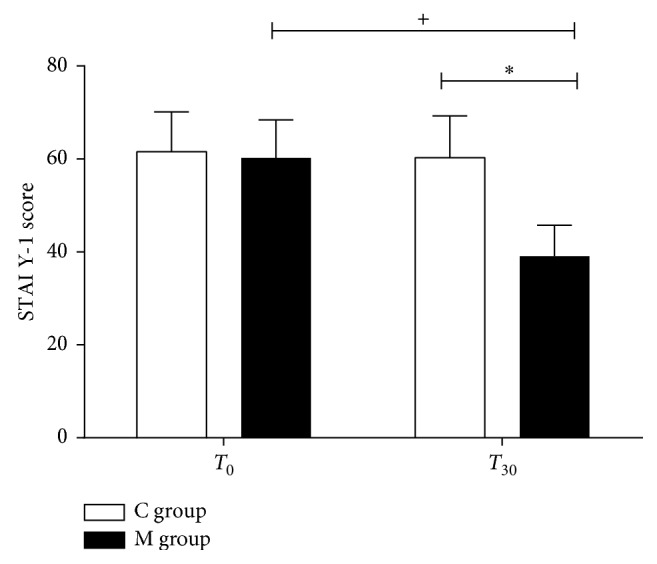
State Trait Anxiety Inventory Questionnaire score. State Trait Anxiety Inventory Questionnaire (STAI Y-1) score in the music group (M group) and control group (C group) at T_0_ and T_30_. Data are presented as mean ± SD; ^+^^,∗^*p* < 0.001.

The baseline STAI Y-1, STAI Y-2, and APAIS scores were similar in both groups (Table 1). The STAI Y-1 score decreased at T_30_ versus baseline only in the M group (39.13 ± 6.7 versus 60.2 ± 8.2, *p* < 0.001), while it remained unchanged in the C group (60.3 ± 9 versus 61.6 ± 9, *p*=0.178). Intergroup analysis showed a difference in M versus C groups at T_30_(*p* < 0.001) (Figure 3).

### 3.2. Analysis of Salivary *α*-Amylase

**Figure 4:**
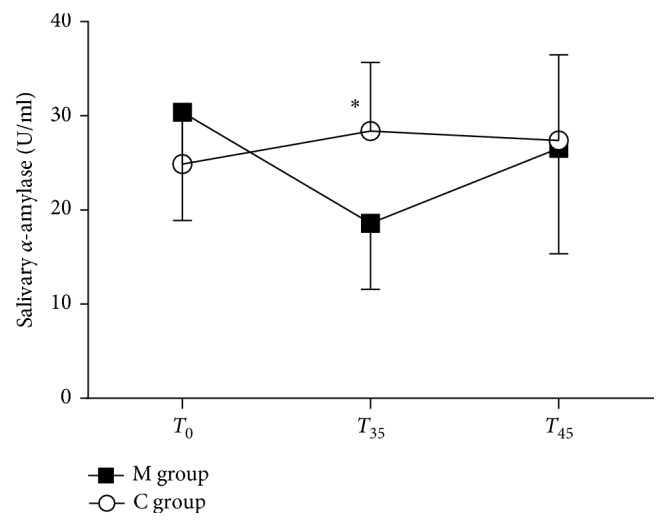
Salivary *α*-amylase levels (U/ml). Salivary *α*-amylase levels (U/ml) in the music group (M group) and control group (C group) at T_0_, T_35_, and T_45_. Data are presented as mean ± SD. ^∗^*p* < 0.001.

Baseline sAA was similar in both groups (M versus C groups: 30.3 ± 11 U/ml versus 24.8 ± 6 U/ml, *p*=0.083). In the M group, the sAA markedly decreased at T_35_ (T_35_ versus T_0_, *p*=0.004; M versus C groups 18.5 ± 7 U/ml versus 28.3 ± 7 U/ml, *p* < 0.001) and slightly increased at T_45_ (T_45_ versus T_0_, *p*=0.5; M versus C groups 26.5 ± 11 U/ml versus 27.3 ± 9 U/ml, *p*=0.810). Conversely, in the C group, the sAA level increased at T_35_ (T_35_ versus T_0_: *p* < 0.001) and slightly decreased at T_45_ (T_45_ versus T_0_: *p*=0.292) (Figure 4).

### 3.3. Analysis of Galvanic Skin Response

Baseline GSR was similar in M and C groups (0.076 ± 0.061 *µ*S versus 0.04 ± 0.051 *µ*S, resp., *p*=0.122). GSR remained unchanged throughout the study period in the M group, while it slightly increased in the C group (T_35_ versus baseline, *p*=0.359).

**Figure 5:**
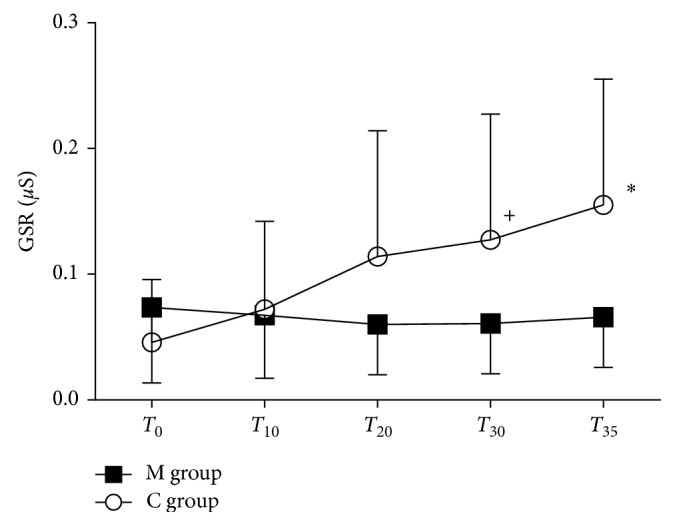
Galvanic skin response. Galvanic skin response (*µ*S) in the music group (M group) and control group (C group) at different time point. Data are presented as mean ± SD. ^+^*p*=0.031 and ^∗^*p* < 0.045.

Intergroup analysis showed a difference between M and C groups at T_30_ (0.064 ± 0.043 *µ*S versus 0.127 ± 0.123 *µ*S, *p*=0.031) and T_35_ (0.068 ± 0.044 *µ*S versus 0.155 ± 0.189 *µ*S, resp., *p*=0.045) (Figure 5).

### 3.4. Analysis of Heart Rate Variability

**Figure 6:**
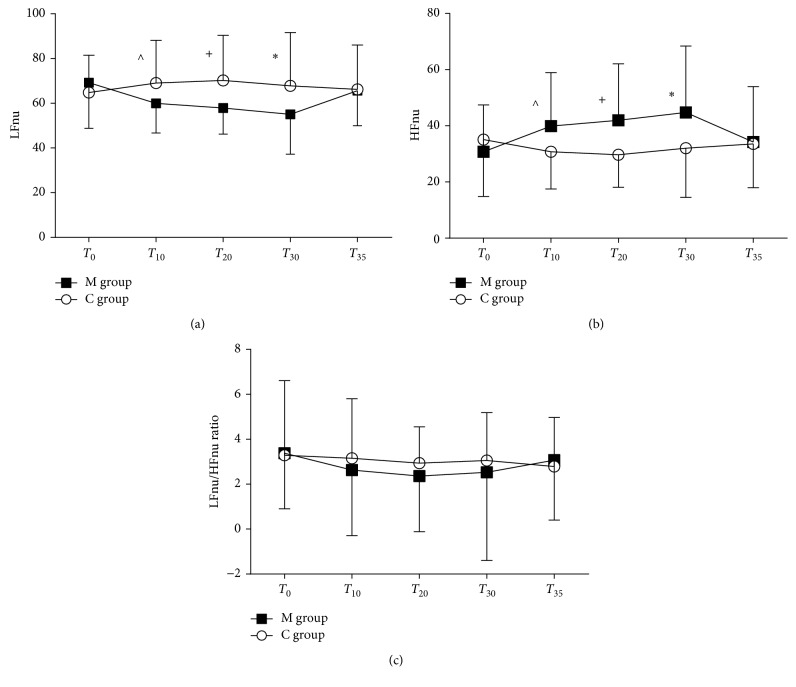
Heart rate variability. (a) Normalized low frequency (LFnu), (b) normalized high frequency (HFnu), and (c) LF/HF ratio in the music group (M group) and control group (C group) at each time point. Data are presented as mean ± SD. ^*p*=0.042, ^+^*p*=0.007, and ∗*p*=0.021.

Baseline LFnu, HFnu, and LF/HF ratio showed similar values in M and C groups (LFnu: 69 ± 16 versus 64 ± 20, resp., *p*=0.813; HFnu: 30 ± 16 versus 35 ± 20, *p*=0.376; LF/HF ratio: *p*=0.908). On T_10_, T_20_, and T_30_, LFnu decreased and HFnu increased in the M group versus C group (LFnu and HFnu *p*=0.042, *p*=0.007, and *p*=0.021 at T_10_, T_20_, and T_30_, resp.) (Figures 6(a) and 6(b)).

In the M group, LFnu progressively reduced during the observation time, especially at T_20_ and T_30_ versus T_0_ (*p*=0.025 and *p*=0.045, resp.) and returned to baseline at T_35_; HFnu significantly increased at T_20_ and T_30_ versus T_0_ (*p*=0.025 and *p*=0.045, resp.). In the C group, LFnu and HFnu trends remained constant during the study (Figures 6(a) and 6(b)).

Mean LF/HF ratio slightly reduced in the M group but not significantly during the time as compared to the C group (Figure 6(c)).

## 4. Discussion

The main results of our study are that, in the group listening to Tibetan music preoperatively, a marked improvement of all anxiety measurements was observed: (a) STAI Y-1 score of anxiety reduced, (b) salivary *α*-amylase levels reduced, (c) GSR trend was constant, and (d) LFnu reduced and HFnu increased during the study period.

To our knowledge, this is the first study examining the effect of Tibetan music on preoperative anxiety, by simultaneous evaluation of the cognitive-emotional, the endocrine, and the autonomic responses by physiological and psychological tests.

Recently, it was demonstrated that allowing patients to listen to the classical or selected favorite music is effective in alleviating acute stress caused by an acute-short term stress factor especially in hospitalized patients and in patients waiting for surgery [15, 28–31]. Furthermore, providing a choice of music was considered a critical factor in lowering anxiety, promoting relaxation, and pain relief [15].

Tibetan music is different from traditional music and classical harmony played by string, flute, or piano, which are usually recommended as therapeutic music [32]. In our study, we used music composed with low frequencies, a constant sonority, and a regular rhythm which may help the relaxation and meditation [33]. The present data showed that subjective anxiety level, as measured by the STAI test, decreased only in patients listening to Tibetan music. Interestingly, this finding was similar to those studies using preferred relaxing music or classical music [12], especially if we consider the Yilmaz et al. investigation showing a lower STAI score in the patients who chose their preferred music versus premedicated patients with midazolam [10].

A series of studies found a correlation between psychological behavior and sAA, assessing the reliability of sAA as an easy to use and noninvasive sympathetic biomarker for stress evaluation [34, 35].

Similarly, we showed that the sAA levels decreased at T_35_ only in patients listening to Tibetan music. Conversely, no sound in the control group increased the sAA levels, suggesting a stressful condition. Interestingly, the impact of various musical genres has important implications in the autonomic response. Nater et al. showed an increased secretion of sAA in men listening to heavy metal music [36].

One explanation might be that the sAA response to stress is complex, since both branches of the ANS are involved in maintaining the oral homeostasis depending upon saliva and its content of proteins. Whereas sAA is secreted from the salivary glands under sympathetic activity, the parasympathetic impulses stimulate the saliva secretion [37].

The focus on the physiological monitoring of anxiety is indicative of the medical concern about the impact of stress on safe anesthesia. Although galvanic skin monitoring has long been considered a measure of physiological and mental stress, a recent Cochrane review showed only one study that analyzed the skin conductivity variations in preoperative anxiety [38]. The authors concluded that anxiety reduction in the patient-selected music group was not reflected in the GSR [39].

Accordingly, we observed a constant GSR trend in the M group.

More recent evidence suggests that HRV is gaining recognition as a measure of cardiac parasympathetic activity and, therefore, an indicator of a relaxation response other than a sensitive indicator of autonomic dysfunction such as in alcoholics, diabetic or cardiopathic patients [40–43]. Previous studies, supported by a recent meta-analysis, revealed that the activation of the ANS in anxiety disorders, can lead to an increase in LF and decrease in HF [12, 38, 44].

Although there is ongoing debate as to interpret HRV [24, 39], there is a large consensus that LF power is modulated by baroreflexes with a combination of sympathetic and parasympathetic activity, whereas HF reflects primary parasympathetic activity [45–47]. LF/HF ratio has a more difficult interpretation, since its use in evaluating the sympathovagal balance has been recently questioned. Many researchers have regarded the notion of sympathovagal balance as merely simplification of a relationship which appears to be more complex than was previously thought. [24, 45].

In this study, normalization of LF and HF has been applied in an attempt to better quantify modulation of the parasympathetic and sympathetic branches of the ANS [40, 48].

Our HRV results appeared consistent with the other cognitive and endocrine results: over the course of the study, an evident decrease in LFnu and an increase in HFnu were observed only in the M group. Furthermore, it is difficult to compare our findings with the classical or preferred music studies regarding to the frequency range due to the scarce information available in the literature [32, 49]. Tibetan music had immediate effects and seems contingent on listening timing which could represent a limit for longer time interval between preoperative preparation and surgery. However, we chose headphones to deliver the music according to the literature, although Lee et al. demonstrated that broadcast is also effective [12, 14]. These overall findings indicate that Tibetan music reduced preoperative stress and may be considered a good strategy for patients waiting for surgery without premedications [12].

Another limit is that the C group had to wear headphones with no sound in order to reduce methodological bias [38, 39]. Even though the severity of self-reported anxiety remained unchanged in this group, we cannot simply exclude that wearing headphones with no sound could have had some effect on their stress level.

Therefore, future studies need to investigate the anxiolytic effect of Tibetan music in perioperative setting by physiological and psychological tools, especially in patients needing emergency surgery.

In conclusion, by combining cognitive and somatic data with endocrine and autonomic response, our results suggest that listening to Tibetan music could help patients to manage preoperative anxiety. The implementation of music in the perianesthesia setting is a noninvasive intervention, easy to administer, and should be considered for clinical practice.

## Figures and Tables

**Table 1:** Demographic and psychological tests.

	Music group	Control group	*p* value
Age (years)	55.1 ± 8.9	58.2 ± 7.7	0.096
*Sex*			
Males/females	15/15	15/15	1
ASA II/III	21/9	19/11	0.6
*Urologic surgery*			0.08
RRP	10	8	
PCNL	14	8	
Others	6	14	
STAI Y-1 T_0_	60.2 ± 8	61.6 ± 9	0.25
STAI Y-2 T_0_	38.9 ± 9	35.9 ± 9	0.10
APAIS T_0_	21.2 ± 5	20.7 ± 4	0.34

Data are expressed as mean ± SD. RRP = radical retropubic prostatectomy; PCNL = percutaneous nephrolithotomy; STAI = State Trait Anxiety Inventory Questionnaire; APAIS = Amsterdam Preoperative Anxiety and Information Scale.

## Abbreviations

ANS: Autonomic nervous system
sAA: Salivary alpha-amylase
HRV: Heart rate variability
GSR: Galvanic skin response
ASA: American Society of Anesthesiologists (ASA)
STAI: State Trait Anxiety Inventory Questionnaire.

## Supplementary Materials

Supplementary Materials Supplementary Material S1: time-frequency of Tibetan music.

## References

[1] Badner N. H., Nielson W. R., Munk S., Kwiatkowska C., Gelb A. W. (1990). Preoperative anxiety : detection and contributing factors. *Canadian Journal of Anaesthesia*.

[2] Leinonen T., Leino-Kilpi H. (1999). Research in peri-operative nursing care. *Journal of Clinical Nursing*.

[3] Morris L. W., Engle W. B. (1981). Assessing various coping strategies and their effects on test performance and anxiety. *Journal of Clinical Psychology*.

[4] Kirschbaum C., Hellhammer D. H. (1994). Salivary cortisol in psychoneuroendocrine research: recent developments and applications. *Psychoneuroendocrinology*.

[5] Gozansky W. S., Lynn J. S., Laudenslager M. L., Kohrt W. M. (2005). Salivary cortisol determined by enzyme immunoassay is preferable to serum total cortisol for assessment of dynamic hypothalamic-pituitary-adrenal axis activity. *Clinical Endocrinology*.

[6] Goldstein D. S. (2003). Catecholamines and stress. *Endocrine Regulations*.

[7] Davis E. P., Granger D. A. (2009). Developmental differences in infant salivary alpha-amylase and cortisol responses to stress. *Psychoneuroendocrinology*.

[8] Task force of the European Society of Cardiology and the North American Society of Pacing and Electrophysiology (1996). Heart rate variability: standards of measurement, physiological interpretation, and clinical use. *Circulation*.

[9] Fenz W. D., Epstein S. (1967). Gradients of physiological arousal in parachutists as a function of an approaching jump. *Psychosomatic Medicine*.

[10] Yilmaz E., Ozcan S., Basar M., Basar H., Batislam E., Ferhat M. (2003). Music decreases anxiety and provides sedation in extracorporeal shock wave lithotripsy. *Urology*.

[11] Beccaloni A. M. (2011). The medicine of music: a systematic approach for adoption into perianesthesia practice. *Journal of PeriAnesthesia Nursing*.

[12] Lee K. C., Chao Y. H., Yiin J. J., Chiang P. Y., Chao Y. F. (2011). Effectiveness of different music-playing devices for reducing preoperative anxiety: a clinical control study. *International Journal of Nursing Studies*.

[13] Tan Y. Z., Ozdemir S., Temiz A., Celik F. (2015). The effect of relaxing music on heart rate and heart rate variability during ECG GATED-myocardial perfusion scintigraphy. *Complementary Therapies in Clinical Practice*.

[14] Nilsson U. (2008). The anxiety- and pain-reducing effects of music interventions: a systematic review. *AORN Journal*.

[15] Mitchell L. A., Macdonald R. A. R. (2006). An experimental investigation of the effects of preferred and relaxing music listening on pain perception. *Journal of Music Therapy*.

[16] Bunt L., Stige B., Storr A. (2014). Music therapy: an art beyond words. *BMJ*.

[17] Fredriksson A. C., Hellstrom L., Nilsson U. (2009). Patients’ perception of music versus ordinary sound in a postanaesthesia care unit: a randomised crossover trial. *Intensive and Critical Care Nursing*.

[18] Spielberger C. (1983). *Manual for the State-Trait Anxiety Inventory (STAI)*.

[19] Spielberger C. D., Gorsuch R. L., Lushene P. R., Vsagg P. R., Jacobs A. G. (1983). *Manual for the State-Trait Anxiety Inventory (Form Y) (self-Evaluation Questionnaire)*.

[20] Julian L. J. (2011). Measures of anxiety: state-trait anxiety inventory (STAI), beck anxiety inventory (BAI), and hospital anxiety and depression scale-anxiety (HADS-A). *Arthritis Care and Research*.

[21] Vitasari P., Wahab M. N. A., Herawan T., Othman A., Sinnadurai S. K. (2011). Re-test of state trait anxiety inventory (STAI) among engineering students in Malaysia: reliability and validity tests. *Procedia-Social and Behavioral Sciences*.

[22] Moerman N., Oosting H. (1996). The amsterdam preoperative anxiety and information scale (APAIS). *Anesthesia and Analgesia*.

[23] Goebel S., Kaup L., Mehdorn H. M. (2011). Measuring preoperative anxiety in patients with intracranial tumors: the Amsterdam preoperative anxiety and information scale. *Journal of Neurosurgical Anesthesiology*.

[24] Von Rosenberg W., Chanwimalueang T., Adjei T., Jaffer U., Goverdovsky V., Mandic D. P. (2017). Resolving ambiguities in the LF/HF ratio: LF-HF scatter plots for the categorization of mental and physical stress from HRV. *Frontiers in Physiology*.

[25] Tarvainen M. P., Niskanen J. P., Lipponen J. A., Ranta-aho P. O., Karjalainen P. A. (2014). Kubios HRV-heart rate variability analysis software. *Computer Methods and Programs in Biomedicine*.

[26] Andre D., Pelletier R., Farringdon J. (2006). *The Development of the SenseWear^®^ armband, a Revolutionary Energy Assessment Device to Assess Physical Activity and Lifestyle*.

[27] Kinetic S. A., Assay R. (2012). *Technical Bulletin Running Multiple Amylase Strips*.

[28] Lee K. S., Jeong H. C., Yim J. E., Jeon M. Y. (2015). Effects of music therapy on the cardiovascular and autonomic nervous system in stress-induced university students: a randomized controlled trial. *Journal of Alternative and Complementary Medicine*.

[29] Kushnir J., Friedman A., EhrenFeld M., Kushnir T. (2012). Coping with preoperative anxiety in cesarean section: physiological, cognitive, and emotional effects of listening to favorite music. *Birth*.

[30] Wang S. M., Kulkarni L., Dolev J., Kain Z. N. (2002). Music and preoperative anxiety: a randomized, controlled study. *Anesthesia and Analgesia*.

[31] Hole J., Hirsch M., Ball E., Meads C. (2015). Music as an aid for postoperative recovery in adults: a systematic review and meta-analysis. *The Lancet*.

[32] Cunningham M. F., Monson B., Bookbinder M. (1997). Introducing a music program in the perioperative area. *AORN Journal*.

[33] Pereira C. (2016). Frequencies of the buddhist meditative chant. *International Journal of Science and Research (IJSR)*.

[34] Noto Y., Sato T., Kudo M., Kurata K., Hirota K. (2005). The relationship between salivary biomarkers and state-trait anxiety inventory score under mental arithmetic stress: a pilot study. *Anesthesia and Analgesia*.

[35] Rashkova M. R., Ribagin L. S., Toneva N. G. (2012). Correlation between salivary alpha-amylase and stress-related anxiety. *Folia Medica*.

[36] Nater U. M., Abbruzzese E., Krebs M., Ehlert U. (2006). Sex differences in emotional and psychophysiological responses to musical stimuli. *International Journal of Psychophysiology*.

[37] Proctor G. B., Carpenter G. H. (2007). Regulation of salivary gland function by autonomic nerves. *Autonomic Neuroscience*.

[38] Bradt J., Dileo C., Shim M. (2013). Music interventions for preoperative anxiety (review). *Cochrane Database of Systematic Reviews*.

[39] Guo J., Wang J. (2005). Study on individual music intervention to reduce preoperative anxiety on patients undergoing laparoscopic surgery. *Chinese Journal of Nursing*.

[40] Kleiger R. E., Stein P. K., Bigger J. T. (2005). Heart rate variability: measurement and clinical utility. *Annals of Noninvasive Electrocardiology*.

[41] Malpas S. C., Whiteside E. A., Maling T. J. (1991). Heart rate variability and cardiac autonomic function in men with chronic alcohol dependence. *Heart*.

[42] Malpas S. C., Maling T. J. B. (1990). Heart-rate variability and cardiac autonomic function in diabetes. *Diabetes*.

[43] Wolf M. M., Varigos G. A., Hunt D., Sloman J. G. (1978). Sinus arrhythmia in acute myocardial infarction. *Medical Journal of Australia*.

[44] Chalmers J. A., Quintana D. S., Abbott M. J.-A., Kemp A. H. (2014). Anxiety disorders are associated with reduced heart rate variability: a meta-analysis. *Frontiers in Psychiatry*.

[45] Appel M. L., Berger R. D., Saul J. P., Smith J. M., Cohen R. J. (1989). Beat to beat variability in cardiovascular variables: noise or music?. *Journal of the American College of Cardiology*.

[46] Billman G. E., Dujardin J. P. (1990). Dynamic changes in cardiac vagal tone as measured by time-series analysis. *American Journal of Physiology-Heart and Circulatory Physiology*.

[47] Furlan R., Guzzetti S., Crivellaro W. (1990). Continuous 24-hour assessment of the neural regulation of systemic arterial pressure and RR variabilities in ambulant subjects. *Circulation*.

[48] Burr R. L. (2007). Interpretation of normalized spectral heart rate variability indices in sleep research: a critical review. *Sleep*.

[49] Thoma M. V., La Marca R., Brönnimann R., Finkel L., Ehlert U., Nater U. M. (2013). The effect of music on the human stress response. *PLoS One*.

