# Supplementary figures and images for: Effects of Tibetan Music on Neuroendocrine and Autonomic Functions in Patients Waiting for Surgery: A Randomized, Controlled Study
Source: Anesthesiol Res Pract. 2018 Mar 5;2018:9683780. doi: 10.1155/2018/9683780 (PMC5859866; doi:10.1155/2018/9683780)

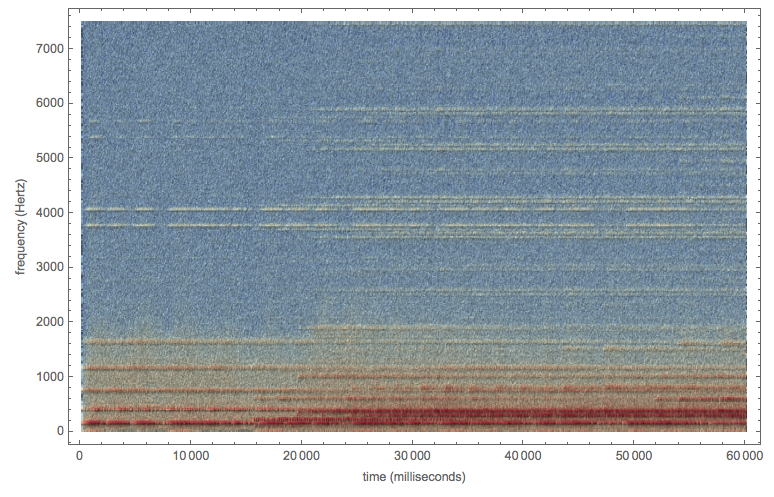

Supplement: Supplementary Materials — Supplementary Material S1: time-frequency of Tibetan music. [file 9683780.f1.png]
